# Management of Second Trimester Fetal Demise in a Noncommunicating Uterine Horn

**DOI:** 10.1155/2015/927037

**Published:** 2015-06-03

**Authors:** R. Tyler Hillman, Homer G. Chin, Sheila K. Mody

**Affiliations:** Department of Reproductive Medicine, UCSD Medical Center, 200 W. Arbor Drive, San Diego, CA 92103, USA

## Abstract

Müllerian anomalies are uncommon but when present they can increase the risk of obstetrical complications. Anomalies such as bicornuate and unicornuate uterus can also increase the surgical risks of pregnancy termination. The diagnostic approach and surgical management must be individualized for each patient so that the termination procedure is safe and preserves fertility. We present a case of a patient with a 17-week pregnancy with fetal demise in a noncommunicating right uterine horn. Laparotomy and hysterotomy were required for evacuation of the fetus. The use of appropriate imaging studies to diagnose suspected uterine anomalies and a flexible and individualized operative strategy are essential for reducing complications associated with the termination of abnormal or unintended pregnancies in women with Müllerian anomalies.

## 1. Introduction

As many as 10% of women in an unselected population have developmental anomalies of Müllerian structures [1]. Müllerian anomalies such as unicornuate uterus, bicornuate uterus, and uterine didelphys are caused by defects in the formation or fusion of bilateral paramesonephric structures and these defects increase the risk of early pregnancy loss, preterm birth, and fetal malpresentation [1]. Müllerian anomalies are usually not detectable by clinical examination and diagnosis often requires pelvic imaging. Even minor Müllerian anomalies may increase the complexity and morbidity associated with the surgical management of abnormal or unintended pregnancy, especially when unrecognized [2, 3]. In the extreme case of pregnancy in a noncommunicating uterine horn, safe pregnancy management depends upon early and accurate diagnosis to prevent complications such as uterine rupture [4, 5]. We present a case of a patient with a second trimester fetal demise in a noncommunicating uterine horn.

## 2. Case Presentation

A 19-year-old G1P0 female with a fetal demise at 17 weeks and 4 days of gestational age presented to the emergency department (ED) with light vaginal bleeding. An ultrasound examination performed one week prior to her presentation demonstrated a nonviable fetus in the right horn of a suspected bicornuate uterus. The patient was referred for dilation and evacuation and was awaiting her appointment at the time of her ED visit. In the ED the patient was hemodynamically stable and was not actively bleeding. Since dilation and evacuation were already planned for the following day, an attempt was made to insert osmotic cervical dilators into the cervical canal under ultrasound guidance. A cervical dilator was easily advanced into the left uterine horn; however, the dilator could not be advanced into the horn that contained the fetus. The procedure was therefore discontinued.

**Figure 1:**
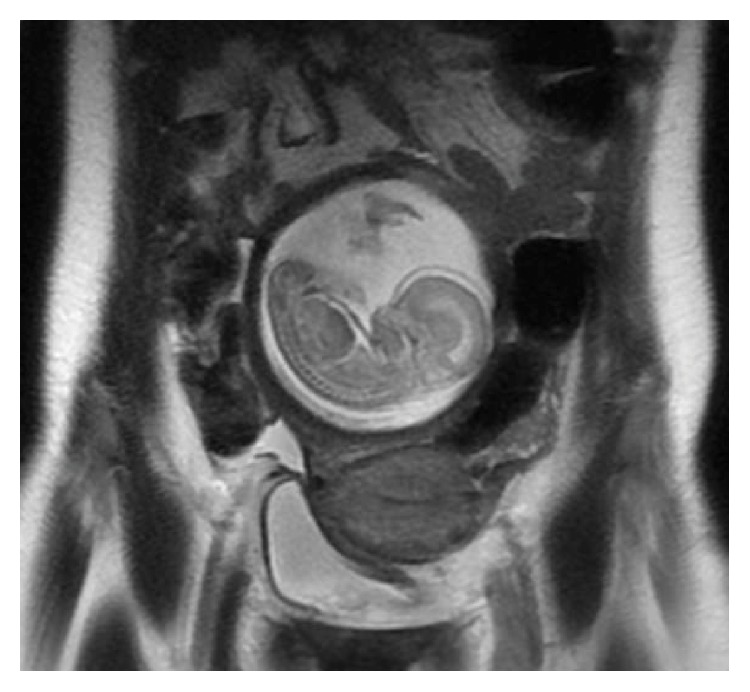
Preoperative MRI showing fetus in right uterine horn with no visible channel between left and right uterine horn.

A pelvic MRI was ordered and results did not reveal a communication between the cervix and the gravid right uterine horn (Figure 1). Although MRI has limitations in the diagnosis of rudimentary uterine horns, it is among the most accurate noninvasive diagnostic modalities [6]. The patient was counseled that the fetus could not be removed via dilation and evacuation. We recommended laparotomy with hysterotomy and the patient consented to proceed.

**Figure 2:**
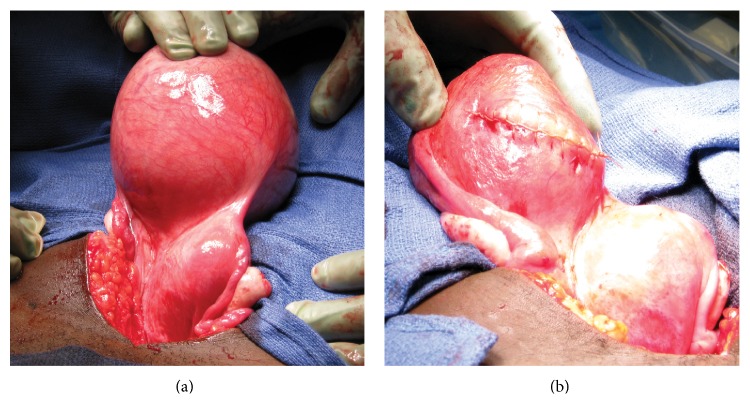
The gross appearance of the bicornuate uterus during surgery: (a) before uterine evacuation, with fetus* in situ*, and (b) after removal of the fetal remains and hysterotomy closure.

At surgery, an infraumbilical vertical midline skin incision was made and exploration of the pelvis revealed an enlarged right uterine horn (Figure 2(a)). Hysterotomy was performed with a transverse incision and the fetus was evacuated with placenta, without difficulty. Palpation of the uterine cavity did not reveal communication with either the cervical canal or the left uterine horn. The hysterotomy was closed in three layers with delayed absorbable suture. Following evacuation and contraction of the right uterine horn, the external aspect of the uterus appeared bicornuate (Figure 2(b)). The abdominal incision was closed.

**Figure 3:**
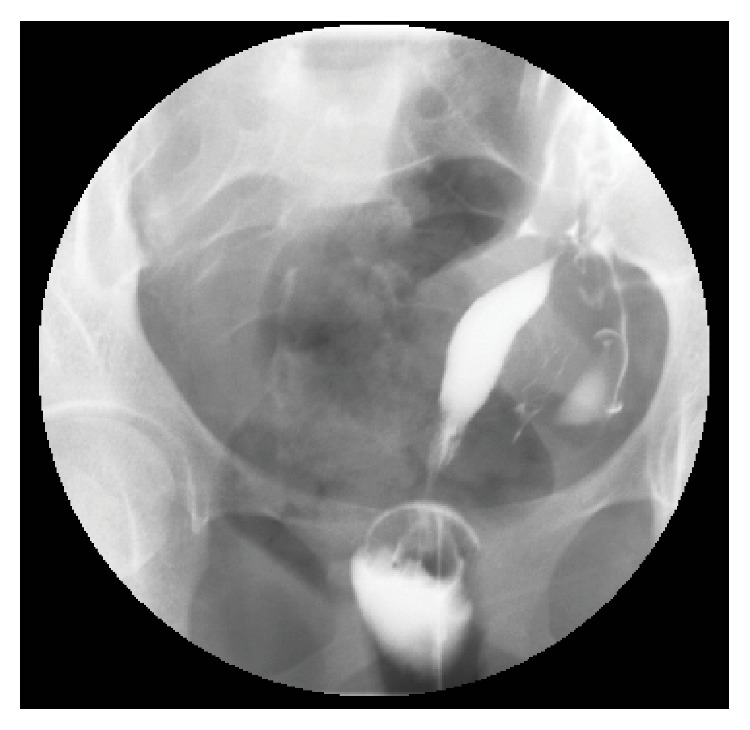
A hysterosalpingogram demonstrating contrast extravasation through the left uterine horn and fallopian tube.

Pathologic examination revealed an anatomically normal fetus. The patient was discharged home after an uneventful postoperative recovery. The patient underwent insertion of an etonogestrel implant (Nexplanon) at her postoperative appointment. Three months after her surgery, the patient underwent hysterosalpingography (HSG), which revealed a left unicornuate uterus without communication with the right uterine horn (Figure 3).

## 3. Discussion

If products of conception are not obtained during surgical management of fetal demise or pregnancy termination the presence of a uterine anomaly must be considered. Bedside ultrasound may not be sufficient to exclude the presence of a uterine anomaly [7]. In the case of a noncommunicating uterine horn, insertion of cervical dilators may increase the risk of uterine perforation. When difficulty is encountered, the use of additional imaging modalities such as MRI should be considered.

Pregnancy in a noncommunicating uterine horn is rare (1 in 76,000 to 1 in 150,000 live births), making this an unusual diagnosis in women presenting for pregnancy termination [4, 8]. When diagnosed in the nonemergent setting, pregnancy in a rudimentary uterine horn should be considered ectopic and managed as an abnormal pregnancy due to the high risk of uterine rupture as well as other maternal complications [4]. Hysterotomy to evacuate a fetus in an anomalous uterus should be considered for advanced gestations and when alternative approaches fail [3, 9].

The absence of communication between this patient's external cervix and the gravid uterine horn, as confirmed by postevacuation HSG (Figure 3), suggests the possibility that fertilization was the result of transperitoneal sperm migration which may account for as many as 50% of pregnancies occurring in women with such anomalies [10]. Removal of the rudimentary horn is therefore recommended because of the high likelihood of recurrent pregnancy in this location [3, 4]. Removal of the rudimentary horn was deferred in this case until the absence of a cervical communication could be confirmed by HSG.
